# Diet supplementation with phytase and xylanase on laying performance, egg quality, apparent metabolizable energy, and phosphorous use in laying hens

**DOI:** 10.14202/vetworld.2025.155-161

**Published:** 2025-01-22

**Authors:** Autchara Kayan, Sombat Prasongsook, Theerawit Poeikhampha

**Affiliations:** Department of Animal Science, Faculty of Agriculture, Kasetsart University, Ngam Wong Wan, Bangkok, 10900, Thailand

**Keywords:** egg quality, feed efficiency, laying hen, nutrient digestability, phosphorus utilization, phytase, poultry enzymes, xylanase

## Abstract

**Background and Aim:**

The inclusion of supplementary enzymes, such as phytase and xylanase, in poultry diets enhances nutrient digestibility and reduces excreta output, minimizing environmental impact. This study aimed to evaluate the effects of phytase and xylanase supplementation on laying performance, egg quality, apparent metabolizable energy (AME), and phosphorus utilization in laying hens.

**Materials and Methods:**

A total of 576 laying hens, aged 47 weeks, were randomly assigned to four dietary treatments: (T1) a control diet, (T2) a negative control diet with reduced energy and phosphorus, (T3) T2 supplemented with phytase (50 g/ton) and xylanase (100 g/ton), and (T4) T2 supplemented with phytase and NSP enzyme mix. The study was conducted over 12 weeks. Data on body weight, laying performance, egg quality, AME, and phosphorus utilization were analyzed using a one-way analysis of variance, with statistical significance set at p < 0.05.

**Results:**

No significant differences were observed in hen-day egg production, egg weight, or feed intake across groups. However, enzyme supplementation (T3 and T4) significantly reduced feed costs per kg egg and feed cost per egg compared to the control (p < 0.05). Eggshell strength improved significantly in T3 and T4 groups, while yolk color declined in these groups compared to T1 (p < 0.05). Other quality metrics, such as albumin height and Haugh units, showed no significant differences. Apparent metabolizable energy, phosphorus retention time, and feces scores were not significantly affected by dietary treatments.

**Conclusion:**

Phytase and xylanase supplementation in diets with reduced nutrient content effectively reduced production costs while maintaining laying performance and improving eggshell strength. These findings support the use of these enzymes as cost-efficient tools in poultry production without compromising egg quality.

## INTRODUCTION

At present, exogenous enzymes are crucial tools for improving the environment, reducing feed cost, increasing the nutritional value of feed, improving nutrient digestibility, promoting growth, and enhancing poultry performance. The digestive system of poultry can synthesize enzymes for the digestion of nutrients. Nevertheless, poultry does not have sufficient enzymes to digest fiber; thus, some commercial exogenous enzymes are supplemented in their diets to improve digestion. Exogenous enzymes have been available for the past several years in the feed industry, including phytase, xylanase, ß-glucanases, proteases, lipases, and galactosidases, and they are expressed in various commercial systems (plants and microorganisms) [1]. Most enzymes in feed diets are utilized as non-starch polysaccharides (NSP) in ingredients such as barley, wheat, rye, and triticale [2]. Phytase supplementation has been used to improve phosphorous utilization in ruminant and monogastric animals, thereby reducing environmental problems associated with organic phosphate excretion [3]. Phytase supplementation in broiler diets reduced phosphate levels and maintained growth performance and skeletal development [4]. Moreover, increasing phytase levels represented an increase in Ca content in the tibia bone on 21 days of age in broilers [5]. Phytase supplementation in a diet with 0.20, 0.25, and 0.30% non-phytate phosphorus (NPP) increased egg production and improved eggshell thickness. Moreover, it influences blood hematology and hormone levels in laying hens. The addition of phytase to the diet containing 0.20, 0.25, and 0.30% NPP increased the plasma concentrations of albumin (ALB), high-density lipoprotein, phosphorus (P), and plasma follicle-stimulating hormone, plasma calcium (Ca), estradiol-17β, and luteinizing hormone [6]. Xylanase was used to break down complex fibrous compounds in feed to utilizable sugar molecules such as NSP. Supplementation with xylanase enhanced body weight gain and decreased feed consumption and feed conversion ratios in broilers. Furthermore, xylanase supplementation improved the digestibility of nutrients, including Ca, phosphorus, dry matter, crude protein (CP), starch, and gross energy [7]. In laying hens, xylanase supplementation did not significantly affect egg production indices and egg quality (except for yolk color) [8].

A previous study by Moita and Kim [9] indicated that feed enzymes, particularly phytase and xylanase, may have advantages for the intestinal health and microbiota of broiler chickens. The hydrolysis of phytic acid and β-1,4-xylan bonds is catalyzed by xylanase and phytase, respectively. Supplementing these enzymes will help to reduce the anti-nutritional properties of phytic acid and NSP, which can improve the intestinal health of broiler chickens. A previous study by Habib [10] revealed that using combined supplementation of phytase, xylanase, and protease enzymes with low 150 kcal of ME/kg and 10% of CP in broiler diets during all growing phases reached the same growth performance, increased carcass, breast meat yield, thigh meat, and economic benefits compared with control broiler chicks. Walker *et al*. [11] studied the combination of these two enzymes (phytase and xylanase) in laying hens. The results revealed that the amount of phosphorus in the excreta of the control group was significantly higher than that of the mixed enzyme phytase and xylanase groups. Phosphorus retention was significantly higher in the mixed enzyme phytase and xylanase groups. Supplementation with phytase in a laying hen diet increased hen-day production, daily egg mass, and P digestibility with increasing levels of phytase. Xylanase supplemented with phytase at 300 phytase units (FTU)/kg improved feed efficiency. Moreover, a diet without phytase and xylanase reduced dry matter and Ca digestibility [12].

Therefore, this study aimed to determine the influence of diet phytase and xylanase supplementation on laying performance, egg quality, apparent metabolizable energy (AME), and phosphorus utilization in laying hens.

## MATERIALS AND METHODS

### Ethical approval

This study was approved by Kasetsart University Institutional Animal Care and Use Committee (ACKU 64-AGR-015).

### Study period and location

This study was conducted from October 2021 to March 2022 at Animal Research Farm, Department of Animal Science, Faculty of Agriculture, Kasetsart University, Thailand.

### Experimental animals

47-week-old laying hens (n = 576) were randomly allocated to four dietary treatments with six replications per treatment and 24 hens per replication. The treatment groups were classified into four treatments, including T1, T2, T3, and T4. The nutrient in the control diet (T1) was developed with mono-dicalcium phosphate as a phosphorous source. (T2) Dietary energy (metabolizable energy) and available phosphorus were reduced by 125 kcal/kg and 0.145%, respectively, and were presented as the negative control diet. (T3) T2 supplemented with phytase (50 g/ton of feed) and xylanase (100 g/ton of feed). (T4) T2 supplemented with phytase and NSP Enz. All treatments were expected to enhance digestion and recovery of nutrients. Phytase (50 g/ton of feed) and xylanase (100 g/ton of feed) were supplemented according to a commercial recommendation. Diets were expressed according to the recommendations of the National Research Council for layers (Table 1). The study period was 12 weeks (48–59 weeks of age). During the experimental period, laying hens were provided with feed and water *ad libitum* and exposed for 16 h:8 h (light: dark). The laying performance, egg quality, AME, and phosphorus utilization were analyzed. The descriptive statistics of laying performance, egg quality, AME, and phosphorus utilization are presented in Table 2.

**Table 1:** Composition of experimental diets.

Ingredients	T1	T2	T3	T4
Corn	49.550	47.311	47.311	47.493
Rice bran oil	2.401	1.000	1.000	1.000
Rice solvent bran	3.000	7.121	7.121	6.994
Cassava meal	4.600	7.000	7.000	7.000
Soybean meal 47.5%	22.365	20.396	20.396	20.408
DDGS (27% CP)	5.000	5.000	5.000	5.000
Pork meal (50% CP)	2.000	2.000	2.000	2.000
Liquid DL-Methionine 88%	0.221	0.186	0.186	0.186
Monodicalciumphosphate22	1.136	0.253	0.253	0.225
CaCo_3_ (limestone powder)	4.038	4.016	4.016	3.975
CaCo_3_ (limestone granular)	5.000	5.000	5.000	5.000
Salt	0.279	0.292	0.292	0.293
Choline chloride 60%	0.100	0.100	0.100	0.100
Mold inhibitor	0.050	0.050	0.050	0.050
Antioxidant	0.010	0.010	0.010	0.010
Vitamins and minerals PX	0.250	0.250	0.250	0.250
Phytase for T3	-	-	0.005	-
Xylanase	-	-	0.010	-
Phytase for T4	-	-	-	0.006
NSP Enz	-	-	-	0.010
Corn cob	-	0.016	0.001	-
Total	100.000	100.000	100.000	100.000
ME for poultry (Cal/kg)	2,750.000	2,624.131	2,750.000	2,750.000
Protein (%)	17.500	17.080	17.500	17.500
Fat (%)	4.870	3.440	3.440	3.446
Fiber (%)	4.000	4.712	4.712	4.702
Calcium (%)	3.900	3.755	3.900	3.900
Total phosphorus content (%)	0.711	0.562	0.707	0.704
Available P for poultry (%)	0.400	0.255	0.400	0.400
Salt (%)	0.350	0.350	0.350	0.350
Lysine (%)	0.863	0.832	0.849	0.849
Methionine+cystine (%)	0.730	0.691	0.730	0.730
Methionine (%)	0.461	0.428	0.432	0.432
Threonine (%)	0.651	0.631	0.664	0.664
Tryptophan (%)	0.195	0.187	0.206	0.206
Choline (mg/kg)	600.000	600.000	600.000	600.000

1% feed was used as a carrier for Enz supplementation. T1=Control diet, T2=Negative control diet with reduced energy and phosphorus, T3=T2 supplemented with phytase (50 g/ton of feed) and xylanase (100 g/ton of feed), T4=T2 supplemented with was supplemented with phytase and NSP Enzyme. CP=Crude protein, NSP=Non-starch polysaccharides, DDGS=Distillers dried grains with solubles, ME=Metabolizable energy, DL=D and L isomer of methionine, PX=Vitamins and minerals premix

**Table 2:** Descriptive statistics of laying performance, egg quality, apparent metabolizable energy, and phosphorus utilization in laying hens.

Parameters	Mean ± SD
Initial body weight (g)	1,904.08 ± 128.98
Final body weight (g)	1,888.98 ± 146.73
Body weight change (g)	−15.17 ± 43.40
Feed intake (g/hen/day)	120.18 ± 2.86
Hen-day egg production (%)	86.07 ± 4.75
Egg weight (g)	68.04 ± 1.93
Egg mass (g)	58.56 ± 2.24
Feed cost/kg egg (Baht)	14.71 ± 0.41
Feed cost/egg (Baht)	1.71 ± 0.06
Shell breaking strength (N)	38.86 ± 2.05
Yolk color	8.77 ± 0.33
Albumin height (mm)	8.98 ± 0.34
Albumin height (Haugh units)	91.77 ± 1.84
Yolk weight ratio (%)	24.09 ± 0.31
Albumin weight ratio (%)	66.19 ± 0.41
Shell weight ratio (%)	9.71 ± 0.29
Yolk: Albumin ratio	36.37 ± 0.71
Shell thickness (mm)	0.397 ± 0.01
Apparent metabolizable energy (kcal/kg)	2,595.33 ± 155.17
Retention time (min)	262.92 ± 11.77
Feces score	3.15 ± 0.50

SD=Standard deviation

### Laying performance

Hen-day egg production rate (%), egg weight (g), egg mass (g), feed intake (g/hen/day), and feed cost per egg were recorded every 2 weeks.

### Egg quality

The eggs were randomly collected at the end of every 2 weeks. Eggshell strength and thickness, egg yolk color, and Haugh units (HU) were measured. Eggshell strength was measured using a Texture Systems Compression Test Cell (model T2100C, Food Technology Co., Ltd., Rockville, MD, USA) and expressed as units of compression force exposed to units of eggshell surface area (kg/cm^2^). Eggshell thickness was defined as the mean value of measurements at three different locations on the egg (air cell, equator, and sharp end) and was measured using a dial pipe gauge (Model 7360, Mitutoyo Co., Ltd, Kawasaki, Japan). Egg yolk color was measured using a Roche color fan (Hoffman-La Roche Ltd., Basel, Switzerland), with 1 = Light pale and 15 = Dark orange. The ALB height was measured using a micrometer (Model S-8400; Ames, Waltham, MA, USA). Albumin and yolk weights were measured using an electronic scale (g). HU were determined using the following equation: HU = 100 log_10_ (H-1.7 W^0.37^ + 7.56), H is the albumin height (mm), and W is the egg weight (g).

### Apparent metabolite energy and phosphorus utilization

The AME was determined using the following formula:

Apparent metabolization energy = GE_diet_ (GE_exceta/digesta_ × [marker_diet_/marker_excreta/digesta_])

GE = Gross energy.

The retention time was measured at the end of the trial period. All chickens were fed 0.5% chromic oxide as an external marker. When the feces turned green, this was recorded as the beginning time. When the feces returned to their normal color, this was recorded as the finishing time.

The feces scores were divided into five grades based on the stability of the feces. Grade 5 describes the highest-rated solids, and Grade 1 begins the lowest stability. There were four contributors scoring at the same time.

### Statistical analysis

The statistical analysis for this study was conducted using one-way analysis of variance (ANOVA) with the SAS software package (SAS Institute Inc., Cary, NC, USA). Significant differences among treatment groups were determined using Duncan’s multiple comparison test within the PROC GLM procedure. A significance level of p < 0.05 was used to identify statistically significant differences. Results are reported as least squares means with their associated standard errors, ensuring an accurate representation of variability within the experimental groups.

## RESULTS

### Laying performance

The initial body weight of laying hens, feed intake, hen-day egg production, egg weight, and egg mass were not significantly different between the treatment groups (p > 0.05). The final body weight of laying hens significantly differed between the treatment groups (p < 0.001). There was a significant difference between treatment groups in feed cost per kg egg and feed cost per egg (p < 0.05). T1 group was represented in the highest final body weight (Table 3). The final body weights of T1, T2, T3, and T4 treatment groups were 1,928.38 ± 134.41, 1,857.43 ± 154.83, 1,890.44 ± 136.56, and 1,879.53 ± 152.61, respectively. Body weight changes were significantly different between T1 and T2 (p < 0.05). T1 group had higher body weight change than T2 group. The body weight changes in T1, T2, T3, and T4 treatment groups were 25.13 ± 30.75, –46.06 ± 58.27, –20.84 ± 13.81, and –19.21 ± 31.06, respectively. Enzyme supplementation (T3 and T4) decreased feed cost per kg egg and feed cost per egg. Moreover, enzyme supplementation significantly improved the feed cost/kg egg and feed cost/egg of hens compared with the T1 group. Enzyme supplementation saved 1.48 and 1.27 baht/kg egg or 0.09 and 0.10 baht/egg in T3 and T4, respectively.

**Table 3:** Influence of xylanase and phytase supplementation in diet on body weight, feed intake, and laying performance at 48–59 weeks of laying hens.

Parameters	T1	T2	T3	T4	p-value
Initial body weight (g)	1,904.02 ± 115.68	1,903.84 ± 147.20	1,911.35 ± 125.37	1,897.13 ± 126.56	0.832
Final body weight (g)	1,928.38 ± 134.41^a^	1,857.43 ± 154.83^b^	1,890.44 ± 136.56^b^	1,879.53 ± 152.61^b^	<0.001
Body weight change (g)	25.13 ± 30.75^a^	−46.06 ± 58.27^b^	−20.84 ± 13.81^ab^	−19.21 ± 31.06^ab^	0.026
Feed intake (g/hen/day)	118.69 ± 2.97	121.63 ± 4.10	120.61 ± 1.80	119.78 ± 2.55	0.346
Hen-day egg production (%)	86.95 ± 1.38	85.70 ± 2.94	86.51 ± 1.36	85.14 ± 2.79	0.518
Egg weight (g)	68.31 ± 2.41	67.81 ± 1.86	68.34 ± 1.27	67.59 ± 1.40	0.854
Egg mass (g)	58.90 ± 3.32	57.45 ± 2.67	59.35 ± 1.24	58.56 ± 0.98	0.528
Feed cost/kg egg (Baht)	21.17 ± 0.91^a^	20.43 ± 0.76^ab^	19.99 ± 0.33^b^	19.94 ± 0.49^b^	0.045
Feed cost/egg (Baht)	1.76 ± 0.08^a^	1.70 ± 0.06^ab^	1.67 ± 0.03^b^	1.66 ± 0.04^b^	0.043

^a,b^Means with different superscripts in the same row indicate a significant difference (p < 0.05.). T1=Control diet, T2=Negative control diet with reduced energy and phosphorus, T3=T2 supplemented with phytase (50 g/ton of feed) and xylanase (100 g/ton of feed), T4=T2 supplemented with was supplemented with phytase and NSP enzyme

### Egg quality

During the experimental periods, albumin height, HU, yolk weight ratio, albumin weight ratio, shell weight ratio, yolk: albumin ratio, and eggshell thickness were not significantly different between the treatment groups (p > 0.05) (Table 4). However, there were significant differences in eggshell strength and yolk color (p < 0.05). The T3 and T4 groups had higher eggshell strength than the T1 and T2 groups. The eggshell strengths of the T1, T2, T3, and T4 treatment groups were 37.48 ± 1.80, 38.52 ± 1.10, 40.86 ± 1.97, and 38.57 ± 1.91, respectively. T2, T3, and T4 had lower yolk color scores than T1. Moreover, yolk color in the T1, T2, T3, and T4 treatment groups were 9.09 ± 0.17, 8.49 ± 0.31, 8.74 ± 0.28, and 8.75 ± 0.31, respectively.

**Table 4:** Influence of xylanase and phytase supplementation in diet on egg quality at 48–59 weeks of laying hens.

Parameters	T1	T2	T3	T4	p-value
Eggshell strength (N)	37.48 ± 1.80^b^	38.52 ± 1.10^b^	40.86 ± 1.97^a^	38.57 ± 1.91^a^	0.021
Yolk color	9.09 ± 0.17^a^	8.49 ± 0.31^b^	8.74 ± 0.28^b^	8.75 ± 0.31^b^	0.011
Albumin height (mm)	8.91 ± 0.21	9.05 ± 0.50	8.95 ± 0.23	9.00 ± 0.41	0.912
Albumin height (Haugh units)	91.34 ± 0.90	92.40 ± 2.84	91.47 ± 1.25	91.88 ± 2.06	0.773
Yolk weight ratio (%)	24.06 ± 0.27	24.12 ± 0.29	24.00 ± 0.33	24.17 ± 0.38	0.818
Albumin weight ratio (%)	66.25 ± 0.33	66.26 ± 0.35	66.24 ± 0.43	66.00 ± 0.54	0.688
Shell weight ratio (%)	9.70 ± 0.13	9.63 ± 0.08	9.78 ± 0.13	9.81 ± 0.27	0.251
Yolk: Albumin ratio	36.31 ± 0.57	36.41 ± 0.62	36.25 ± 0.74	36.53 ± 1.00	0.925
Shell thickness (mm)	0.395 ± 0.01	0.393 ± 0.00	0.4000 ± 0.01	0.398 ± 0.01	0.344

^a,b^Means with different superscripts in the same row indicate a significant difference (p < 0.05.). T1=Control diet, T2=Negative control diet with reduced energy and phosphorus, T3=T2 supplemented with phytase (50 g/ton of feed) and xylanase (100 g/ton of feed), T4=T2 supplemented with was supplemented with phytase and NSP enzyme

### Apparent metabolite energy and phosphorus utilization

There were no significant differences between treatment groups for AME, retention time, and feces score (p > 0.05) (Table 5). The AME of the T1, T2, T3, and T4 treatment groups were 2,647 ± 163.62, 2,540.20 ± 155.58, 2,581.75 ± 192.22, and 2,561.72 ± 133.93. The retention times in the T1, T2, T3, and T4 treatment groups were 256.83 ± 11.44, 266.83 ± 11.25, 263.50 ± 15.49, and 264.50 ± 8.55. The feces scores of the T1, T2, T3, and T4 treatment groups were 3.33 ± 0.47, 3.08 ± 0.26, 3.17 ± 0.54, and 3.00 ± 0.71.

**Table 5:** Influence of xylanase and phytase supplementation in diet on apparent metabolizable energy, retention time, and feces scores at 59 weeks of age in laying hens.

Parameters	T1	T2	T3	T4	p-value
Apparent metabolizable energy (kcal/kg)	2,647.67 ± 163.62	2,540.20 ± 155.58	2,581.75 ± 192.22	2,561.72 ± 133.93	0.694
Retention time (min)	256.83 ± 11.44	266.83 ± 11.25	263.50 ± 15.49	264.50 ± 8.55	0.522
Feces score	3.33 ± 0.47	3.08 ± 0.26	3.17 ± 0.54	3.00 ± 0.71	0.719

T1=Control diet, T2=Negative control diet with reduced energy and phosphorus, T3=T2 supplemented with phytase (50 g/ton of feed) and xylanase (100 g/ton of feed), T4=T2 supplemented with was supplemented with phytase and NSP enzyme

## DISCUSSION

Supplementing xylanase and phytase will help reduce the anti-nutritional properties of phytic acid and NSP, which can improve the intestinal health of chickens [9], increase hen-day production, daily egg mass, and P digestibility with increasing levels of phytase [12]. This study investigated the influence of xylanase and phytase supplementation on body weight, feed intake**,** and laying performance. Diet supplementation with xylanase and phytase significantly affected the final body weight and body weight change of laying hens. Supplement enzyme levels decreased in final body weight and body weight change. This study showed that the control diet group was fed more rice bran oil than the other groups. This will lead to the direct accumulation of abdominal fat and a high increase in body weight. The initial body weight of laying hens, feed intake, hen-day egg production, egg weight, and egg mass were not significantly different between the groups. The overall weight gain was not affected by the addition of phytase and/or xylanase. Hen-day production increased with the addition of phytase [12]. The addition of protease, xylanase, and phytase boosted broiler body weight gain until day 21 and the feed conversion ratio, which gradually decreased until the end of the grower phase. In young broilers, the addition of exogenous enzymes to the diet was more significant [13]. Xylanase decreases apparent dry matter digestibility, reduces digesta viscosity, and improves nutritional digestibility [14].

Egg quality in this study revealed that xylanase and phytase supplementation affected eggshell strength and yolk color. Supplement enzymes increased eggshell strength and decreased yolk color score. Albumin height, HU, yolk weight ratio, albumin weight ratio, shell weight ratio, yolk: albumin ratio, and eggshell thickness were not significantly different between the groups. The supplementation with phytase or xylanase influenced egg quality at the end of the experiment. The interaction between phytase × xylanase was detected for dry matter (DM) and Ca digestibility. This might be because the measured Ca content was larger than expected; in that case, providing the bird with sufficient Ca to form eggshells would not further benefit from increased Ca availability [12]. A diet supplemented with phytase showed increased bone mineralization. Both enzymes resulted in stronger bone, as evidenced by increased bone mineralization [15]. Phytase improves the utilization of P and other nutrients, such as Ca and amino acids. Phytase supplementation in a diet with 0.20, 0.25, and 0.30% NPP increased egg production and improved eggshell thickness. This might be an increase in phytase levels represented by increased Ca content in tibial bone that leads to Ca accumulation in bone to synthesize eggshell [5, 6]. Dietary phytase had no effect on egg quality, including eggshell strength, eggshell color, egg yolk color, and HU. Adding up to 10,000 FTU/kg of phytase to laying hen diets did not improve their productivity [16]. The rate of egg production increased at 20,000 FTU/kg phytase [16]. The relationship between minerals and xylanase in diet and the significant increase in eggshell thickness as the amount of dietary xylanase increased in weeks 3, 6, 9, and 12 of age. The use of minerals may be related to the efficiency of xylanase in enhancing eggshell thickness [17]. Xylanase improves the performance of laying hens. Egg production was lower in birds fed the high-soluble NSP diet without xylanase than in those fed other treatments. In addition, xylanase supplementation decreased yolk weight at 32 weeks of age and an increase in yolk color score at 28 and 32 weeks of age. The ability of xylanase to decrease bile salt de-conjugation, increase fat digestion, and reduce digesta viscosity enhances nutrient digestion and releases carotenoids. It may be responsible for the observed increase in yolk color [14]. The production performance of hens aged between 24 and 33 weeks was unaffected by the addition of various multi-enzyme sources to mixed diets, and no changes in egg quality, except for index and egg shape [18].

This study revealed that enzyme supplementation did not influence AME, retention time, and feces score. In agreement with a previous study revealing that xylanase supplementation did not affect nitrogen retention [19]. In broilers, however, enzyme supplementation increased the AME throughout the starter and growth phases and enhanced nitrogen retention during the starter phase [13]. Increasing concentrations of phytase in laying hen diets may increase phytate P degradation and ileal P digestibility and minimize P excretion. Consequently, phytase degrades that dietary can increase phosphorous availability and digestibility while lowering fecal P excretion. This is a significant factor in environmental contamination [20].

## CONCLUSION

This study evaluated the effects of dietary supplementation with phytase and xylanase enzymes on laying performance, egg quality, apparent metabolizable energy, and phosphorus utilization in laying hens. The findings revealed that enzyme supplementation, particularly in the T3 (phytase and xylanase) and T4 (phytase and NSP enzymes) groups, significantly reduced feed costs and improved eggshell strength without compromising overall laying performance or egg quality. These results underscore the economic and practical viability of using phytase and xylanase in laying hen diets to enhance productivity and reduce production costs.

The study’s robust experimental design, involving a substantial sample size (576 hens) and multiple replicates for each dietary treatment, ensures statistical reliability and validity. Comprehensive parameters, including laying performance, egg quality, and nutrient digestibility, provide a holistic understanding of the impact of enzyme supplementation. The use of a control and negative control group further strengthens the comparative insights.

Poultry producers should consider incorporating phytase and xylanase into diets with reduced nutrient content to enhance cost efficiency without compromising production performance. Focus should be placed on optimizing enzyme dosages based on specific production goals, such as improving eggshell strength or reducing feed costs. Enzyme supplementation can be a key component of sustainable poultry farming practices to mitigate environmental phosphorus pollution.

However, the study was limited to hens aged 47–59 weeks, which may not reflect the effects of enzyme supplementation across other age groups. It did not assess the long-term impact of enzyme supplementation on overall flock health or environmental phosphorus excretion. The study also relied on specific enzyme dosages and may not account for variations in dietary composition or management practices across different production systems.

Future research should extend to include hens at different stages of their laying cycle to evaluate age-specific benefits of enzyme supplementation. Investigations should also focus on the long-term impacts of enzyme supplementation on flock health, nutrient retention, and environmental sustainability. Exploring the synergistic effects of phytase and xylanase with other feed additives, such as proteases, may further optimize feed utilization. In addition, field trials under commercial production conditions are essential to validate laboratory findings and assess economic feasibility on a larger scale.

This study contributes valuable insights into the application of dietary enzymes in poultry nutrition, paving the way for more sustainable and economically viable practices in egg production.

## AUTHORS’ CONTRIBUTIONS

AK and TP: Conducted the study, data analysis, and drafted and revised the manuscript. SP: Provided technical assistance during the experiments. TP: Study conception and design and reviewed the manuscript. All authors have read and approved the final manuscript.
